# Assessing Critical Care Nurse's Knowledge and Adherence to Evidence-Base Guidelines for Ventilator-Associated Pneumonia Prevention in Palestinian Hospitals

**DOI:** 10.1155/nrp/1434479

**Published:** 2024-12-27

**Authors:** Ismael Ahmad Al-Nawaja'a, Basma Salameh, Dalia Toqan, Bahaaeddin M. Hammad, Imad Fashafsheh

**Affiliations:** ^1^Critical Care of Nursing Intensive Care Unit Department, Yatta Governmental Hospital, Yatta, Hebron, State of Palestine; ^2^Department of Nursing, Arab American University of Jenin, Jenin, State of Palestine

**Keywords:** adherence, EBGs, ICUs, knowledge, VAP

## Abstract

**Background:** Patients in critical care units who are connected to mechanical ventilators (MV) often face the risk of ventilator-associated pneumonia (VAP). Therefore, the aim of current study is to describe critical care nurses' knowledge and adherence to evidence-base guidelines (EBGs) for preventing the occurrence of VAP.

**Methodology:** A cross-sectional study was applied. Data were collected through a self-administered questionnaire completed by all critical care nurses (*n* = 213) working at Palestinian hospitals. Descriptive and inferential statistic was utilized to describe ICU nurse's knowledge and adherence to VAP prevention EBGs.

**Results:** The study revealed that the mean knowledge score for critical care nurses was (50.8%). Overall, the findings indicated that nurses' knowledge of VAP guidelines was at an average level. A statistically significant difference (*p* value = 0.049) in the knowledge level was observed based on nurses' qualifications. On the other hand, the study found that nurses' adherence to EBGs was an acceptable (mean = 8.3, 69.2%). No substantial differences in adherence level were identified based on respondents' characteristics.

**Conclusion:** Critical care nurses possess an average level of knowledge regarding EBGs for preventing VAP, alongside demonstrating an acceptable level of adherence to these guidelines.

**Summary**

• It provides guidance for implementing targeted educational interventions and quality improvement initiatives aimed at enhancing patient safety and reducing VAP incidence in Palestine hospitals, focusing on critical care knowledge and adherence to EBGs.

## 1. Background of the Study

Patients in critical care units who are connected to mechanical ventilators (MV) often face the risk of ventilator-associated pneumonia (VAP) [1]. The incidence rate of VAP infection ranges between 9% and 67%, with high mortality rates ranging between 13% and 94%. Approximately 90% of pneumonia cases observed in the ICU occur in patients connected to MV [2, 3]. The incidence rate of VAP increases for patients who are intubated, ranging from 6% to 21%, compared to other patients. This increase is associated with prolonged periods of ventilation on the MV [2, 3]. VAP develops in 28% of patients who received on MV, as the rate of occurrence varies according to the MV period [4]. A study by [5] found the incidence of VAP among patients on MV ranging from 25.9% to 26.7% per 1000 ventilator days. These results align with those reported worldwide, indicating that VAP stands as a prevalent healthcare-associated infection [6, 7]. Another study conducted in Malaysia found that 30% of patients on MVs developed VAP [8]. In critical care units, the incidence of VAP ranges from 25% to 42%, making it the second largest infection [9, 10]. Additionally, among patients receiving mechanical ventilation, the risk of VAP increases by 5%–65% daily [11].

VAP refers to pneumonia that develops after the ventilator has been used for more than 48 h, and without any initial symptoms and signs of pneumonia in the patient prior to ventilation initiation. VAP can significantly prolong the patient's hospital stay and result in increased hospital costs [12].

Controlling avoidable risk factors can significantly reduce the occurrence of VAP, and the incidence of VAP decreases with enhanced knowledge and adherence to evidence-base guidelines (EBGs) for VAP prevention [2]. Previous studies have shown that VAP leads to prolonged MV use, increased antimicrobial usage, elevated risk of mortality, and higher hospitalization costs [13, 14].

The American Thoracic Society, the Center for Disease Control and Prevention, and the Institute for Health Care Improvement have jointly released EBGs targeting the prevention of VAP, aimed at enhancing the quality of care. However, it remains unclear whether nurses adhere to these recommendations. Additionally, the impact of nurses' compliance on the occurrence rate of VAP is also uncertain [15].

Critical care nurses are reported to lack knowledge regarding the VAP prevention bundle [14–17].

Previous studies have found that educating and training nurses on VAP prevention bundles can lead to a decrease in the incidence of VAP and enhance nurses' adherence to preventative measures [18–21]. Moreover [22], emphasized the importance of utilizing EBGs to reduce the incidence of VAP in patients receiving MVs. Nurses can significantly enhance patient outcomes by implementing evidence-based practices for VAP through ongoing education [23, 24]. Intensive care nurses have a vital role in reducing the incidence of VAP by adhering to EBGs while caring for patients connected to ventilators. Monitoring nurses' practices for patients on endotracheal tubes is essential for reducing infection-related complications, shortening hospital stays, and consequently reducing treatment costs [2, 25, 26].

In addition to the limited scientific research on this subject in Palestine, the main aim of the current study is to describe critical care nurses' knowledge and adherence to EBGs for preventing the occurrence of VAP in patients undergoing mechanical ventilation during the treatment plan.

## 2. Aim of the Study

The aim of current study is to describe critical care nurses' knowledge and adherence to EBGs for preventing the occurrence of VAP. Additionally, the study aims to investigate the knowledge levels based on participants' characteristics.

A cross-sectional descriptive study was conducted among all critical care nurses at Palestinian hospitals to investigate their knowledge and adherence to EBGs related to VAP prevention.

### 2.1. Population

The survey was distributed to all nurses working in the ICUs of Palestinian hospitals. The total number of ICUs nurses was 251. Data were collected during the months of April to June 2022.

### 2.2. Sample Size

The sample size depended on response rate of nurses working in the ICUs department. Based on Statdisk online sample calculator (https://www.statdisk.com) the minimal sample size was 135, according to these values:

• Confidence level = 95%
• *P* = the decimal representation of the proportion of choices made (0.25 used for sample size needed)
• *E* = margin of error = ±0.05
• Population = 251.

### 2.3. Instrumentation

Data were collected from ICU nurses using a self-administered questionnaire tailored to describe nurse's knowledge and adherence in preventing the occurrence of VAP. The tool used in this research was adapted and edited with permission from [7, 27], after obtaining approval for its use. It was then further developed to align with the objectives of this study. The original instrument was initially designed to describe nurses' knowledge and adherence to EBGs for VAP prevention. Survey development involved a comprehensive literature review, consultation with the supervisor, and discussions with experts in the fields of anesthesiology and intensive care.

The survey was divided into three sections:

• Section one: Demographic and nursing experience which include (age; gender; years of experience; qualification type).
• Section two: Knowledge of EBGs for Prevention of VAP
• The survey consisted of twelve multiple-choice questions, each with one correct answer, to investigate nurses' knowledge of EBGs for preventing VAP. Additionally, two new questions were added into this section of the survey: (1) use of proton pump inhibitor medications and (2) endotracheal tube cuff pressure measurement.
• The level of knowledge was categorized into four components based on the total percentage scores, as outlined in the study by Aziz et al. [16]: “Excellent = 81%–100%, Good = 61%–80%, Average = 41%–60%, Poor ≤ 40%.”
• Section three: Nurses' Adherence to EBGs for Prevention VAP included 12 items related to nurses' practices. These questions required selecting the most appropriate answer from three options: (1) done completely, (2) not done completely and accurately, and (3) not done. Additionally, two new questions were added to this section: (1) perform sedation vacation daily for sedated mechanically ventilated patients and (2) assess intubated patients daily to determine if they can transition from invasive to noninvasive ventilation.

The level of adherence was categorized into three components based on their percentage scores, as outlined in the study by Al-Sayaghi [28]: Total scores < 50% were considered unsafe adherence, scores between 50% and 75% were considered acceptable adherence, and scores > 75% were considered high adherence.

### 2.4. Inclusion/Exclusion Criteria

The inclusion criteria encompassed all registered nurses employed in adult critical care units in Palestinian hospitals with more than 6 months of experience in critical care units. Exclusion criteria included student nurses and part-time nurses.

### 2.5. Sample and Sampling

The study sample included all nurses in intensive care as well as ICU head nurses. The research sample included all nurses working in ICUs in all public hospitals who meet to inclusion criteria. The number of participants in the research was determined based on the percentage of response rate from all ICUs nurses in governmental hospitals, totaling 252. The achieved response rate was 84.9% (*n* = 213).

### 2.6. Pilot Study

A Pilot study was conducted with the participation of 10 critical care nurses to assess the questionnaire for spelling accuracy, ease of completion, and comprehensiveness. The aim was to identify any linguistic ambiguity and make necessary modifications. Additionally, the questionnaire was reviewed by an intensive care specialist and an anesthesiologist to verify its strength and accuracy, and their valuable recommendations were incorporated.

The questionnaire was designed to be completed within 5–10 min, which was deemed suitable for intensive care nurses who are who are on a busy work schedule.

### 2.7. Validity

After constructing the questionnaire, it was reviewed by five experts, including an Anesthesiologist, an Intensive Care Specialist, two Academic Teachers, and a Clinical Application Specialist. Their comments were carefully considered, and necessary modifications were made before data collection. The survey was recommended by the experts following their review.

### 2.8. Reliability

The questionnaire was comprised a set of comprehensive questions aimed at guiding evidence-based practiced to prevent the occurrence of VAP. The reliability of the questionnaire was assessed using Cronbach's coefficient. For the knowledge section, Cronbach's Alpha was calculated to be 0.92. Similarly, for the adherence section, Cronbach's Alpha was determined to be 0.87.

### 2.9. Ethical Consideration

The study received ethical approval from the Helsinki Committee (The approval number is PHRC/HC/1064/22.), as well as from the Palestinian Ministry of Health, and the hospitals administrators where the study was conducted. Additionally, confidentiality of the participants was strictly maintained, and no names or personal information were disclosed in this study.

### 2.10. Data Analysis

Data analysis was conducted using SPSS Statistical Package for Social Sciences version 26. Inferential statistics were used to investigate potential correlations and differences between the research variables. Descriptive statistics, including frequencies, means, and medians, were used to summarize the survey data. Due to the non-normal distribution of the data, nonparametric tests were selected to analyze the association between the general characteristics of nurses and their knowledge scores. The link between the characteristics of nurses (age, years of experience, qualification, and current role in ICU) and their knowledge of and adherence to EBGs was examined using the Kruskal–Wallis test. Statistical significance was defined as a *p* value < 0.05.

## 3. Results

A total of 99 respondents (46.5%) were younger than 28 years old. The majority of participants reported having less than 5 years of experience in the ICU (*n*=135, 63.4%), and most held a bachelor's degree (*n*=163, 76.5%). Additionally, the majority of respondents did not have a specialized role in the ICU (*n*=191, 89.7%).

### 3.1. Respondents Knowledge

**Figure 1:**
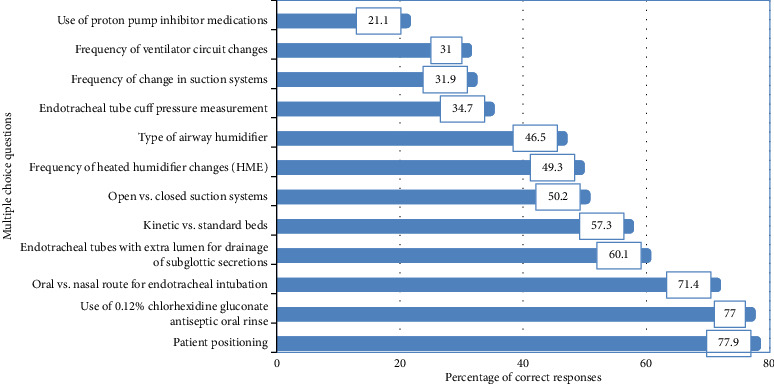
“Percentage of correct responses to the knowledge questions” (*n*=213).

The percentages of participants who provided correct answers to each question are presented in Figure 1. Questions related to patient position, chlorhexidine gluconate antiseptic oral rinse, and route of ventilation had the highest accuracy rates, with percentages of 77.9%, 77.0%,  and 71.4%,  respectively. Conversely, questions regarding the use of proton pump inhibitors, rate of ventilator circuit replacement, and rate of suction system replacement had the lowest accuracy rates, with percentages of 21.1%, 31%, and 31.9%, respectively.

Table 1 displays the level of knowledge among respondents. The majority of nurses demonstrated an average level of knowledge regarding EBGs (54.0%), while only 7% of the participants exhibited an excellent knowledge level. Additionally, 16.9% had good knowledge, and 22.1% had poor knowledge.

The total knowledge scores concerning participants' characteristics are presented in Table 2. The mean score on 12 questions was 6.1(50.8%). Kruskal–Wallis test results indicated no substantial differences in knowledge level between age, years of ICU experience, and the role in ICU. However, a statistically significant difference (*p* value = 0.049) in the knowledge levels of respondents was observed based on qualifications, with nurses holding a master's degree demonstrating the highest means knowledge scores, followed by those with a bachelor's degree then diploma holders.

### 3.2. Respondents Adherence

**Figure 2:**
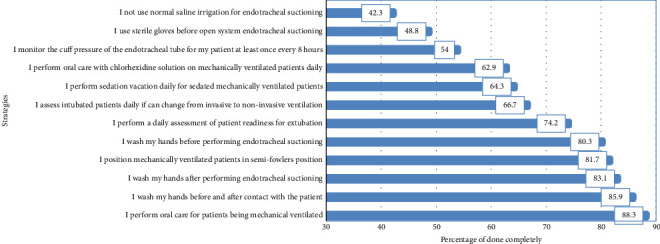
Nurses' adherence to evidence-based guidelines.

Figure 2 illustrates nurses' adherence to EBGs. The procedures with the highest adherence rates were “performing oral care” and “washing hands,” with rates of 88.3% and 85.9%, respectively. Conversely, the practices with the lowest adherence rates were related to “not using normal saline irrigation” and “using of sterile gloves for open endotracheal suctioning,” with rates of 42.3% and 48.8%,  respectively.

Based on the level of adherence categories (where total scores < 50% were considered unsafe adherence, scores between 50% and 75% were considered acceptable adherence, and scores > 75% were considered high adherence), the statistical analysis revealed that 76 respondents showed high adherence, 108 respondents had acceptable adherence, and 29 respondents exhibited unsafe adherence, as presented in Table 3.

Table 4 represents linear regression to predict the extent of the association between knowledge of and self-reported adherence to EBGs. The coefficient of determination (*R*^2^) value of 0.03 suggests that 3% of the variation in the mean adherence scores is explained by the knowledge level. The analysis revealed that knowledge level (per unit increase) was independently correlated with an increase in total adherence score of 0.20 points (95% CI: 0.05 − 0.35). On the other hand, the other variables do not have a significant impact on the mean adherence score for this study.

## 4. Discussion

The study aimed to describe the knowledge and adherence of critical care nurses to EBGs for the prevention of VAP among all nurses working in critical care units in the West Bank, Palestine.

According to the findings of the current study, nurses exhibit an average level of knowledge regarding VAP guidelines.

The questions with the highest accuracy rate concerned patient position, chlorhexidine gluconate antiseptic oral rinse, and route of ventilation (77.9%), (77.0%), and (71.4%),  respectively. Conversely, the questions with the lowest accuracy rate pertained to the use of proton pump inhibitors, rate of changing ventilator circuit, and frequency of changing suction system (21.1%), (31%), and (31%), respectively. These findings are consistent with a study conducted in Australia, which aimed to investigate ICU nurse's knowledge of VAP and adherence to EBGs for VAE prevention. The study reported a median score of 6 (IQR: 5–7) out of 10 for knowledge of EBGs. Among the survey questions, those related to patient positioning received the highest correct answers (90.9%), while questions about equipment use, specifically the use of kinetic beds versus standard, had the lowest correct answers (23%). This highlighting a lack of participants' knowledge in this particular area, with an overall correct response rate of 54.2% [7].

The knowledge score in this study was higher than that of a previous study conducted in Sana'a City, Yemen, in 2018, which disclosed that over 50% of nurses demonstrated poor knowledge regarding EBGs for preventing VAP [29]. Moreover, a study by [16] evaluating ICU nurses' knowledge and utilization of the ventilator care bundle for preventing VAP showed that nurses had insufficient knowledge of the ventilator bundle, as reflected by a mean score of 37.5%. Furthermore, another study conducted among ICU nurses in Kuala Lumpur Hospital revealed that 59.5% of ICU nurses had poor knowledge of EBGs to prevent the occurrence of VAP [17].

The lack of knowledge among critical care nurses concerning EBGs for preventing VAP may be attributed to several factors. These include the challenges of keeping up with changing scientific updates, the absence of educational courses within hospitals to facilitate the reduction of VAP incidence, and a shortage of competent staff [20]. Moreover, existing systemic factors within the healthcare system in Palestine, such as resource scarcity and lack of continuing education, may contribute to this low level of knowledge [30–32].

Regarding adherence, critical care nurses showed higher compliance with conducting oral care on mechanically ventilated patients and practicing hand hygiene. Conversely, lower adherence rates were observed in relation to “not using of normal saline irrigation” and “using sterile gloves for open endotracheal suctioning.” This is consistent with a study indicating higher adherence to oral hygiene and positioning, but lower adherence to endotracheal practices [7].

Another study was published in 2018 aiming to describe critical care nurses' knowledge, adherence, and obstacles related to ventilator bundle in Finland. The study found that the knowledge levels and adherence accounted for 71.1% and 65.8%, respectively, which is consistent with the findings of the current study [33].

A study conducted in Jordan aimed to describe the adherence of Jordanian nurses with EBGs to prevent VAP. The results showed that 45.6% of the nurses expressed “insufficient compliance which is lower than our study” [15].

The adherence to EBGs for preventing the occurrence of VAP in this research is deemed acceptable, especially in light of the limited knowledge of the EBGs among nurses in the ICUs. This indicates that there are experience learned through clinical practice that may not be based on scientific knowledge and evidence, highlighting the need for improvement in aligning practices with evidenced-based knowledge.

In the current study, no substantial differences in knowledge level were found between age, years of ICU experience, and the role in ICU affect. However, there was a significant difference (*p*-value = 0.049) in the knowledge level of respondents based on qualification. This finding is consistent with a study was conducted in 2018 in Addis Abeba, Ethiopia, which aimed to investigate adult intensive care nurses' knowledge, practice, and associated factors on the prevention of VAP. This study showed a significant difference in knowledge between respondents in terms of educational levels (*p* value = 0.04) [12]. However, Madhuvu et al. [7] study showed that there were no significant correlations between the participants' years of experience and their overall knowledge score and adherence to EBGs to decrease risks for VAP. Nevertheless, the study found a substantial significant relationship between the attainment of a postgraduate degree and their overall knowledge score. Another study in Tanzania in 2021 revealed that the total knowledge score was 38.6%. Nurses with a degree or a higher level of nursing education outperformed those with a diploma or a lower level of nursing education significantly more often (*p*=0.004). The average compliance score for EBGs used to prevent VAP was 60.8% [20].

A study conducted to describe the compliance of Jordanian nurses with EBGs for preventing VAP showed that nurses with greater experience and prior education on VAP exhibited higher compliance scores compared to those with less experience and no previous VAP education [15]. However, our current study did not find substantial differences in adherence level based on respondents' characteristics (age, years of ICU experience, qualification, and the current role in ICU).

In addition, a study conducted by Madhuvu et al. [7] showed results consistent with the current study. No correlations were found between following evidence-based recommendations and participants' knowledge, years of experience, specialist titles and postgraduate degrees.

Moreover, a study that amid to describe ICU nurse's adherence to VAP guidelines in middle region of Jordan hospitals revealed an adherence levels of 81.3%. The participants' score of VAP care knowledge was found to be poor, at 43%. Additionally, there was a significant correlation between participants' score on VAP care knowledge and their adherence to VAP guidelines (*p*=0.012), which is consistent with the current study [23]. Similarly, in the study conducted by Jansson et al. [33]; a low correlation was observed between knowledge and adherence scores (*ρ* 0.48) (*p* < 0.001).

Indeed, the extent of knowledge of something enhances commitment to it, as evidenced in the current research where the extent of adherence to EBGs for prevents the occurrence of VAP is affected by the extent of knowledge in them. This can be explained by the notion that providing education for nurses will improve their adherence nurses' adherence levels to EBGs for preventing VAP [34].

### 4.1. Limitation of the Study

The use of self-administered questionnaire can be considered as a limitation of the study, as it relies on participants' self-reporting. Additionally, the study did not consider some factors such as nursing shortage, nurse-patient ratio, and workload, which could be related to ICUs nurses' knowledge and adherence to EBGs to prevent the occurrence of VAP. These factors warrant further research study.

## 5. Conclusion

This study highlights the critical need to enhance ICU nurses' knowledge and adherence to EBGs for preventing VAP. While adherence to EBGs was acceptable, knowledge level was low, emphasizing the need for targeted interventions. Notably, higher qualifications were associated with better knowledge, underscoring the role of advanced education in improving ICU nurse's competency. To address these gaps, the study underscores the importance of continuous in-service education and interventional strategies tailored to ICU settings. Furthermore, incorporating EBGs for VAP prevention into nursing curricula is to equip future nurses with the necessary tools to combat VAP effectively.

### 5.1. Implications for Practice

These results can serve as a basis for reconsidering practical practices in ICUs, with a focus on increasing nurses' awareness of EBGs to reduce the incidence of VAP. Furthermore, dissemination of the current results among ICUs nurses can play a crucial role in raising their level of knowledge and adherence to EBGs to reduce the risk of VAP. Additionally, the limited level of knowledge and adherence observed in the current study suggests the necessity of continuing educational initiatives and the assessment of the EBG implementation for VAP prevention.

### 5.2. Recommendations

Our study recommended holding workshops and educational courses in hospitals to educate health care professionals regarding EBGs aimed at preventing the occurrence of VAP. Additionally, disseminate the current results to hospitals administrators and policymakers, highlighting the importance of implementing these guidelines to prevent of VAP and emphasizing the need to assess the extent of their implementation.

## Figures and Tables

**Table 1:** Knowledge total scores according to respondents.

Knowledge total score	Excellent	Good	Average	Poor
*N* (%)	15 (7.0)	36 (16.9)	115 (54.0)	47 (22.1)

**Table 2:** Nurses' mean knowledge scores (%), median, and IQR by respondent characteristics.

Characteristic	Mean (%)	Median (IQR)	Significance (*p* value)
Age			0.551
<28	6.1(50.8)	6.0(2.0)	
28 − 35	6.0(50.0)	6.0(3.0)	
>35	6.2(51.7)	6.0(2.3)	
Years of ICU experience			0.230
< 5	6.2(51.7)	6.0(3.0)	
5 − 10	5.6(46.7)	6.0(3.0)	
>10	6.3(52.5)	6.0(2.5)	
Qualification			0.049
Diploma	5.5(45.8)	6.0(2.0)	
Bachelor's	6.1(50.8)	6.0(3.0)	
Master's	6.7(55.8)	6.5(2.0)	

*Note:* The *p* value (Kruskal–Wallis test), indicates how score differ across the various subgroups.

**Table 3:** Total adherence scores by respondents.

Adherence total score	High	Acceptable	Unsafe
*n* (%)	76 (35.7)	108 (50.7)	29 (13.6)

**Table 4:** Linear regression analysis of mean adherence to evidence-based guidelines.

Predictor or independent variable	*B* ± standard error	95% CI	(*p* value)	*R* ^2^
Knowledge score	0.20 ± 0.08	0.05 − 0.35	0.01	0.03
Qualification (per category increase)	0.27 ± 0.33	−0.38 − 0.93	0.41	0.003
Age (per category increase)	0.10 ± 0.23	−0.55 − 0.35	0.66	0.001
Years of ICU of experience (per category increase)	0.14 ± 0.23	−0.31 − 0.60	0.61	0.002
Current role in ICU (per category increase)	−0.17 ± 0.09	−0.34 − 0.01	0.08	0.012

## Data Availability

Data are available on request through the authors themselves.
